# Recent Advances in Biosynthesis Technology and Future Functional Foods

**DOI:** 10.3390/foods14213782

**Published:** 2025-11-04

**Authors:** Weigang Liu, Dayong Ren, Fankui Zeng

**Affiliations:** 1 Lanzhou Institute of Chemical Physics, Chinese Academy of Sciences, Lanzhou 730000, China; liuweigang@licp.cas.cn; 2 Academy of Food Science and Engineering, Jilin Agricultural University, Changchun 130117, China; rdy79@163.com

Biosynthesis technology is defined as the application of biological systems—such as microorganisms, cultured cells, or enzymes—to the conversion of simple substrates into high-value biomolecules through engineered metabolic pathways. By integrating metabolic engineering and synthetic biology, this technology offers a sustainable, efficient, and precise alternative to conventional chemical synthesis for the production of functional ingredients in the food, pharmaceutical, and biotechnology sectors [1,2,3]. With the advances in synthetic biology, metabolic engineering, and precision fermentation, this approach supports the sustainable production of bioactive compounds that provide health benefits beyond basic nutrition [4,5,6,7]. The practical implementation of this technology contributes to alleviating resource limitations and minimizing environmental footprints by substituting traditional agricultural extraction and chemical synthesis with sustainable microbial biomanufacturing approaches [5,8,9]. One notable example is the microbial biosynthesis of human milk oligosaccharides (HMOs). HMOs, which are naturally present in breast milk, play a critical role in shaping the infant gut microbiota and supporting immune development. Advances in metabolic engineering and synthetic biology have allowed microorganisms such as *E. coli* and *Saccharomyces cerevisiae* to produce HMOs at scale, providing sustainable and functional ingredients for fortified infant formulas. These advances also support the development of functional foods fortified with targeted nutrients, probiotics, or plant-based compounds, improving nutritional quality and promoting health [3,5,10,11,12]. The integration of biotechnology and nutritional science is thus positioned to direct the evolution of next-generation, evidence-based functional foods [2,6,7,10,12]. This Special Issue compiles six research articles and one review, which collectively focus on functional foods, food nutrition and health, food storage and preservation, and bioactive food ingredients.

Regarding functional foods, Xue et al. optimized the red vinasse-blue round scad functional food. Optimal conditions (2.8 g/g red vinasse, 4 °C, 10 h) produced the best quality. Storage trials showed that microwaving accelerated lipid oxidation, boiling altered fatty acids, and foil-baking caused the greatest fat loss. A partial least squares regression clarified the fatty acid dynamics, supporting strategies for lipid-stable nutraceutical processing—Xue et al. [contribution 1]. In another study, Bacillus velezensis P45 was non-hemolytic, sensitive to 15 antibiotics, and lacked virulence or resistance genes, confirming its safety. An in silico analysis predicted a 99.88% probiotic probability, and phylogeny grouped P45 with known probiotic Bacilli, highlighting it as a promising candidate—da Rosa et al. [contribution 2].

Regarding food nutrition, health, storage, and preservation, Xue et al. developed a pH-responsive bilayer film for yellowfin seabream, featuring an emulsified layer (chitosan/linseed oil) and an indicator layer (carrageenan/gelatin with grape seed anthocyanins and curcumin). Optimized via response surface methodology, the film preserved fish while monitoring freshness, showing a yellow-to-reddish-brown color change that reflected real-time quality—Xue et al. [contribution 3]. Nwaudah et al. proposed a solution to Nigeria’s wheat scarcity and malnutrition by developing biscuits supplemented with oyster mushroom and okara flour. Replacing 20% of the wheat with mushroom powder optimally boosted protein, resulting in a product that maintained stability for two months when baked at 180 °C for 18 min and packaged in carton-in-polyethylene—Nwaudah et al. [contribution 4]. Xue et al. demonstrated that red vinasse effectively preserved the quality of blue round scad during storage by significantly inhibiting lipid oxidation (lower TBARS), retaining PUFAs, and retarding the increases in TVB-N and pH (*p* < 0.05). A shelf-life model based on TBARS data exhibited a high accuracy (R^2^ > 0.95), predicting a significantly extended shelf-life in the treated group, thus supporting the application of red vinasse for scad preservation—Xue et al. [contribution 5].

Regarding bioactive food ingredients, Li et al. isolated a novel neutral polysaccharide from Lycium barbarum, which exhibited a specific backbone/branch structure, semi-crystallinity, and thermal stability. This polysaccharide protected PC12 cells from CoCl_2_-induced hypoxia by scavenging reactive oxygen species (ROS), restoring antioxidant enzyme activity, and regulating associated gene expression, demonstrating its potential for treating ischemic stroke—Li et al. [contribution 6]. Flavonoids, abundant in fruits and vegetables, regulate plant growth, pigmentation, and stress resistance. In humans, compounds such as flavonols and anthocyanins provide anti-inflammatory, neuroprotective, and cardioprotective benefits. Yet, their therapeutic use is constrained by their low bioavailability (5–10% absorption). Enhancing their clinical potential requires deeper insight into molecular mechanisms and improved delivery strategies—Zheng et al. [contribution 7].

This Special Issue presents seven studies showcasing recent advances in biosynthesis technology and functional food research. By leveraging synthetic biology, metabolic engineering, and precision fermentation, biosynthesis enables the sustainable production of bioactive compounds with demonstrable health-promoting effects. In the area of functional foods, one study optimized red vinasse–blue round scad formulations and storage conditions, while another identified Bacillus velezensis P45 as a safe and effective probiotic strain. Regarding nutrition, health, and preservation, a pH-responsive bilayer film was developed for real-time freshness monitoring, and mushroom–okara-enriched biscuits enhanced protein content while providing a practical approach to addressing wheat scarcity. Furthermore, red vinasse effectively preserved fish quality and extended shelf life. Regarding bioactive ingredients, a novel *Lycium barbarum* polysaccharide exhibited neuroprotective effects against hypoxia, and studies on flavonoids highlighted their health benefits despite their limited bioavailability. Collectively, these findings underscore the critical role of biotechnology in advancing next-generation, evidence-based functional foods.
